# Modifying Ligand-Induced and Constitutive Signaling of the Human 5-HT_4_ Receptor

**DOI:** 10.1371/journal.pone.0001317

**Published:** 2007-12-19

**Authors:** Wei Chun Chang, Jennifer K. Ng, Trieu Nguyen, Lucie Pellissier, Sylvie Claeysen, Edward C. Hsiao, Bruce R. Conklin

**Affiliations:** 1 Gladstone Institute of Cardiovascular Disease, University of California at San Francisco, San Francisco, California, United States of America; 2 Institut de Génomique Fonctionnelle, Universités de Montpellier, CNRS UMR 5203, Montpellier, France; 3 INSERM U661, Montpellier, France; 4 Graduate Program in Pharmaceutical Sciences and Pharmacogenomics, University of California at San Francisco, San Francisco, California, United States of America; 5 Department of Medicine, University of California at San Francisco, San Francisco, California, United States of America; 6 Department of Cellular and Molecular Pharmacology, University of California at San Francisco, San Francisco, California, United States of America; Cairo University, Egypt

## Abstract

G protein–coupled receptors (GPCRs) signal through a limited number of G-protein pathways and play crucial roles in many biological processes. Studies of their *in vivo* functions have been hampered by the molecular and functional diversity of GPCRs and the paucity of ligands with specific signaling effects. To better compare the effects of activating different G-protein signaling pathways through ligand-induced or constitutive signaling, we developed a new series of RASSLs (receptors activated solely by synthetic ligands) that activate different G-protein signaling pathways. These RASSLs are based on the human 5-HT_4b_ receptor, a GPCR with high constitutive G_s_ signaling and strong ligand-induced G-protein activation of the G_s_ and G_s/q_ pathways. The first receptor in this series, 5-HT_4_-D^100^A or Rs1 (RASSL serotonin 1), is not activated by its endogenous agonist, serotonin, but is selectively activated by the small synthetic molecules GR113808, GR125487, and RO110-0235. All agonists potently induced G_s_ signaling, but only a few (e.g., zacopride) also induced signaling via the G_q_ pathway. Zacopride-induced G_q_ signaling was enhanced by replacing the C-terminus of Rs1 with the C-terminus of the human 5-HT_2C_ receptor. Additional point mutations (D^66^A and D^66^N) blocked constitutive G_s_ signaling and lowered ligand-induced G_q_ signaling. Replacing the third intracellular loop of Rs1 with that of human 5-HT_1A_ conferred ligand-mediated G_i_ signaling. This G_i_-coupled RASSL, Rs1.3, exhibited no measurable signaling to the G_s_ or G_q_ pathway. These findings show that the signaling repertoire of Rs1 can be expanded and controlled by receptor engineering and drug selection.

## Introduction

Heptahelical G protein–coupled receptors (GPCRs) are the largest family of human cell-surface receptors, encompassing more than 340 hormone receptors and 350–460 olfactory receptors [1], [2]. They are activated by peptide hormones, odorants, photons, biogenic amines, phospholipids, and many other extracellular signals. Upon activation, GPCRs undergo conformational changes that allow active and reversible signaling through a limited number of G-protein pathways (G_s_, G_i_, G_q_, G_12/13_). These signals mediate a wide variety of physiological responses, including heart rate, chemotaxis, cell proliferation, neurotransmission, and hormonal responses. Owing to their physiological importance, GPCRs are of great medical interest. Indeed, they are targets for at least 40% of modern pharmaceuticals [3].

Although many drugs target GPCRs, studies of GPCR signaling *in vivo* have been hampered by the lack of specific agonists and antagonists for many of the receptors. GPCRs display molecular and functional diversity, such as the type of G-protein signaling pathway associations, different levels of constitutive activity, and different signaling responses due to different ligand-selective conformations [4], [5]. This diversity enables the receptors to transmit unique extracellular signals but hampers efforts to sort out the relative contributions of each signaling pathway or the roles of constitutive signaling for each receptor.

To better study the diversity of GPCRs, we developed receptors activated solely by synthetic ligands (RASSLs) by modifying their structures to render them unresponsive to endogenous hormones. Instead, RASSLs are activated by small-molecule drugs [6], allowing them to be used to activate specific G-protein pathways rapidly and reversibly and to mimic the speed, localization, regulation, and amplification of endogenous GPCR signals [7].

Since it is impractical to convert all GPCRs into RASSLs, we and others have focused on representative G_s_-, G_i_-, and G_q_-coupled GPCRs, which stimulate adenylyl cyclase, inhibit adenylyl cyclase, and stimulate phospholipase-C, respectively. Ro1 (RASSL opioid 1), the prototype RASSL based on a G_i_-coupled κ-opioid receptor [8], provided a proof of concept for this strategy. Its G_i_ response to natural ligands is 0.001% of that of the wildtype receptor, but it is potently activated by the synthetic agonist spiradoline [8]–[10]. Ro1 decreases heart rate in mice [11] and affects taste sensation in the tongue [9]. In addition to serving as powerful tools to dissect the G-protein signaling *in vivo*, RASSLs can yield insights into fundamental aspects of receptor diversity [12]. For instance, constitutive signaling of Ro1 led to cardiomyopathy [11], diminished bone formation [13], and induced hydrocephalus [10]. These constitutive signaling phenotypes would have been difficult or impossible to identify by studying endogenous receptors.

Multiple RASSLs have since been made, including a G_s_-coupled RASSL based on the melanocortin-4 receptor [14], a G_q_-coupled RASSL based on the histamine 1 receptor [15], and a series of RASSLS based on muscarinic receptors [16]. These RASSLs are useful tools; however, it is still advantageous to derive a series of RASSLs with distinct G-protein signaling from the same parental GPCR. It can be difficult to compare the effects of RASSLs based on different parental GPCRs since these RASSLs could have different pharmacokinetics, constitutive activity, desensitization kinetics, and cellular localization.

**Figure 1:**
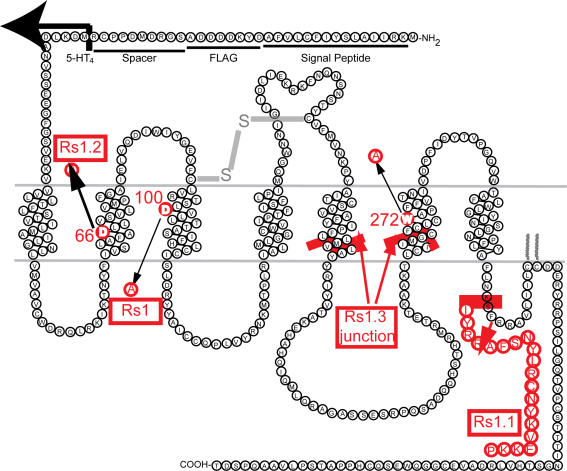
Human 5-HT_4_-based RASSLs. A signal peptide and the FLAG epitope were added to the N-terminus of the human 5-HT_4_ receptor. A D^100^A mutation was introduced by site-directed mutagenesis to create the G_s_-coupled RASSL (Rs1). An additional point mutation (D^66^A, D^66^N, or W^272^A) was added to Rs1 to modulate constitutive signaling. Rs1.1 is the Rs1-C-5-HT_2C_ chimera with enhanced G_q_ signaling. Rs1.2 is Rs1 with an extra D^66^A mutation that decreased constitutive G_s_ signaling. Rs1.3 is the Rs1-i3-5-HT_1A_ chimera with G_i_ signaling. The junctions in Rs1.1 and Rs1.3 indicate ends of domain swapping where the 5-HT_4_ has been replaced by 5-HT_2C_ or 5-HT_1A_, respectively.

To better study GPCRs, we built a series of RASSLs based on the human 5-HT_4_ receptor (Figure 1), which has several advantages over other serotonin receptors. First, its pharmacological properties are well established [17]. Second, its agonists have milder effects (increased gastrokinesis [18], augmented memory acquisition and retention [19], increased chronotropic and inotropic cardiostimulation [20], and enhanced cortisol release [21]) than other serotonergic drugs. Third, it has a large number of synthetic ligands, which allows us to identify differences in their effects on that receptor. Fourth, a single mutation (D^100^A) in the mouse 5-HT_4_ receptor dramatically reduced its affinity for serotonin, its endogenous ligand. This mutation also allows synthetic agonists and antagonists for the wildtype receptor to activate the mutant, turning 5-HT_4_-D^100^A into a RASSL [22].

Finally, we reasoned that novel receptors coupling to other signaling pathways could be created by making chimeras of the 5-HT_4_ receptor with other family members. Altering the G-protein selectivity of GPCRs is often difficult because it is based on receptor conformation determined by multiple regions of the receptor [23]. Changing multiple regions involves large internal mutations that often lead to receptor instability. A better strategy for altering G-protein signaling characteristics is to swap domains between structurally similar receptors within the same family. The 5-HT_4_ receptor belongs to a family of at least 15 receptors, each with different subfamilies that engage different G-protein signaling pathways. The 5-HT_4_, 5-HT_6_, and 5-HT_7_ subfamilies are G_s_ coupled, the 5-HT_1_ subfamily is G_i_ coupled, and the 5-HT_2_ subfamily is G_q_ coupled [24]. These characteristics could expedite our efforts to make purely G_s_-, G_i_-, and G_q_-coupled RASSLs.

Here, we describe a new series of RASSLs developed to modify the ligand-induced and constitutive signaling of the human 5-HT_4_ receptor. These modified GPCRs will help us better study the effect of constitutive G_s_ signaling and ligand-induced G_s_, G_s_/G_q_, and G_i_ signaling *in vivo*.

## Results

### Human 5-HT_4_ D^100^A is a G_s_-coupled RASSL

**Figure 2:**
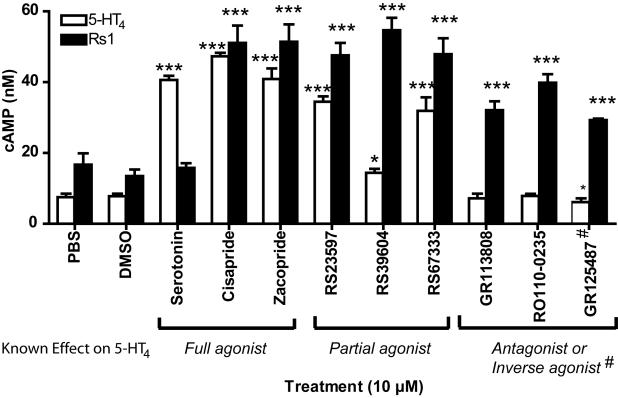
Rs1 is a G_s_-coupled RASSL. Rs1 was efficiently activated by small compounds known to be full agonists (cisapride, zacopride), partial agonists (RS39604, RS67333, and RS23597), antagonists (GR113808, RO110-0235), or inverse agonists (GR125487) for the wildtype 5-HT_4_ receptor. It was not activated by its endogenous agonist (serotonin). Values are mean±SD of three independent experiments in which 25 ng of 5-HT_4_ or Rs1 receptor cDNA was electroporated into 5×10^6^ cells. DMSO, dimethyl sulfoxide.

**Figure 3:**
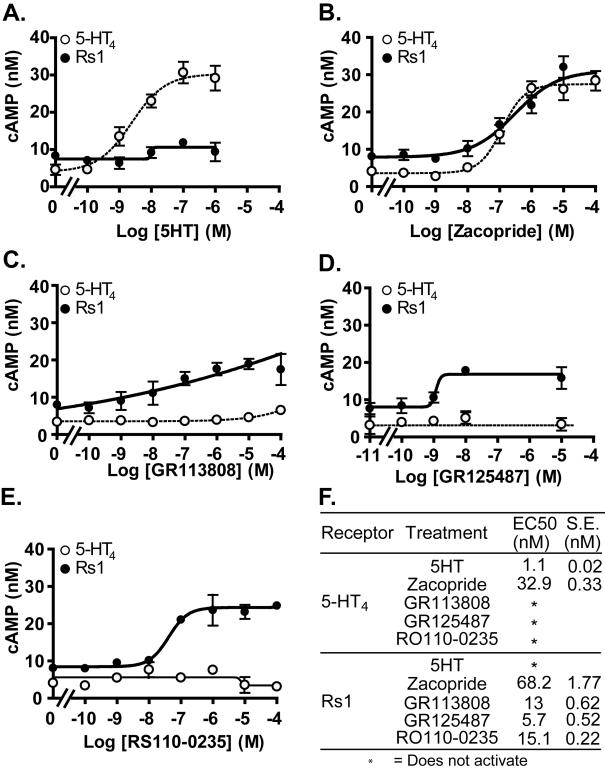
Rs1 activation occurs in the nanomolar range. (A–E) Rs1 transfectants were stimulated with increasing amounts of drugs. The D^100^A mutation in Rs1 makes the receptor insensitive to serotonin. It was efficiently activated by GR113808, GR125487, and RO110-0235, which do not activate the wildtype 5-HT_4_ receptor. Values are mean±standard deviation of three independent experiments in which 25 ng of 5-HT_4_ or Rs1 receptor cDNA was electroporated into 5×10^6^ HEK293 cells. (F) Best-fit estimate of the half-maximal effective concentration (EC50). Values are mean±SEM of three independent experiments.

The D^100^A mutation in the mouse 5-HT_4_ receptor converts it into a RASSL [22], but its effects on the human 5-HT_4_ receptor have not been tested. We now extend these findings to the human 5-HT_4_ receptor (Figure 1). To determine if antagonists for the wildtype 5-HT_4_ receptor also activate the human 5-HT_4_-D^100^A mutant, we tested a variety of compounds. The mutant receptor was not activated by serotonin (Figures 2, 3A), but it was activated by agonists (cisapride, zacopride), partial agonists (RS23597, RS39604, RS67333), antagonists (GR113808, RO110-0235), and an inverse agonist (GR125487) for the wildtype 5-HT_4_ receptor (Figure 2). Interestingly, GR125487 showed a specific response, as demonstrated by the steep concentration-response curve (Figure 3D). In addition, GR113808, GR125487, and RO110-0235 potently activated G_s_ signaling of 5-HT_4_-D^100^A without stimulating the wildtype receptor (Figures 3B–E). The mutant receptor was selectively activated by multiple synthetic ligands (GR113808, GR125487, and RO110-0235) but not serotonin. We named it Rs1 (RASSL serotonin 1) (Figure 1).

### Rs1 has a high level of constitutive signaling

**Figure 4:**
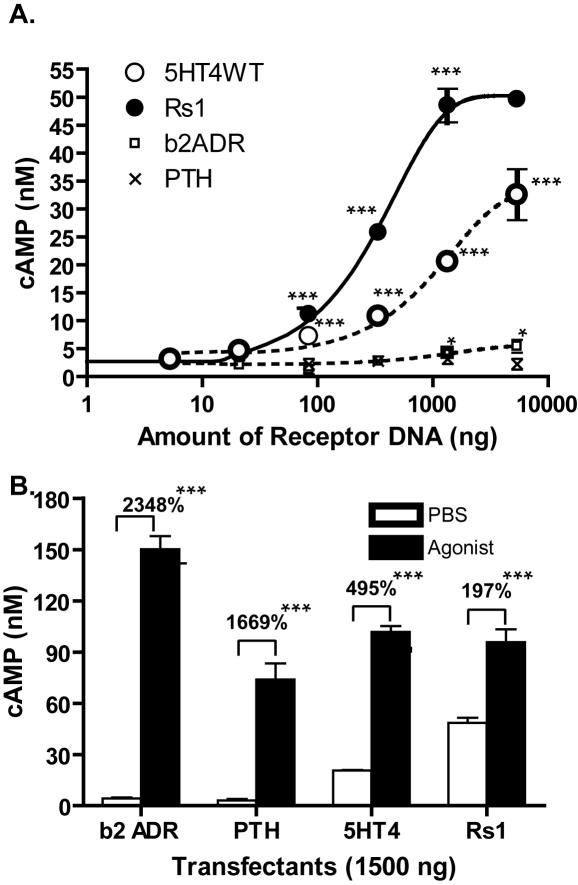
Rs1 has significant constitutive activity. (A) Rs1 and the wildtype 5-HT_4_ receptor both had much higher constitutive activity than the β2-adrenergic receptor (β_2_-ADR) and parathyroid hormone receptor (PTH). ***p<0.005, and * p<0.05 vs. mock-transfected cells (*t* test). (B) Rs1 was still activated when 1.5 µg of receptor cDNA was electroporated into 5×10^6^ HEK293 cells. Rs1 only showed a 197% increase over constitutive signaling. 5-HT_4_ and Rs1 were stimulated with 1 µM of zacopride. β_2_-ADR and PTH were stimulated with 1 µM of isoproterenol and human PTH (1-34) peptide, respectively. Activation of any of the four receptors resulted in significantly higher cAMP over the PBS mock treated samples. ***p<0.005 (*t* test). All experiments were repeated three times. Values are mean±SD.

We next examined the constitutive signaling of Rs1 in more detail. Rs1 showed greater constitutive signaling than the wildtype receptor at all levels of transfection (Figure 4A). Constitutive activity was observable when only 25 ng of receptor cDNA per 5×10^6^ HEK293 cells was transfected (Figure 2). The highest level of constitutive activity, achieved with 5.4 µg of receptor cDNA per 5×10^6^ HEK293 cells, was 1.5 times greater than that of the wildtype 5-HT_4_ receptor (49.6±1.25 nM vs. 32.5±4.04 nM, p<0.005) and >10-fold higher than that of the control receptors (the β_2_-adrenergic and parathyroid hormone receptors), which have low levels of constitutive signaling (Figure 4A). Despite the high level of constitutive signaling, both the 5-HT_4_ receptor and Rs1 could still be further activated by zacopride (Figure 4B).

### Ligand-specific G_q_ signaling in Rs1

**Figure 5:**
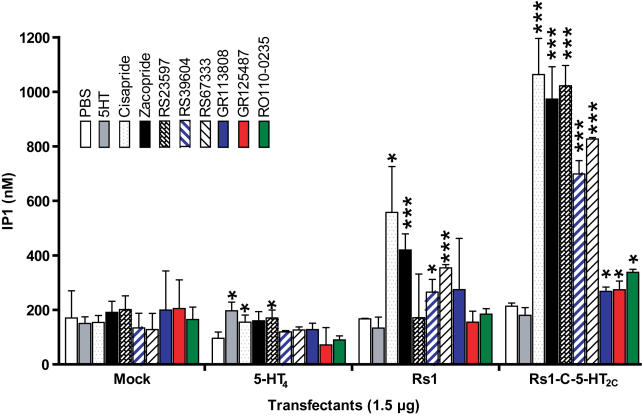
The wildtype 5-HT_4_ receptor, Rs1, and the Rs1-C-5-HT_2C_ chimera exhibit different G_q_ signaling properties. G_q_ signaling was analyzed by measuring the accumulation of inositol 1 phosphate (IP1). The 5-HT_4_ receptor showed increased G_q_ signaling when activated by serotonin, cisapride, and zacopride but not by RS39604 or RS67333, relative to mock treatment with HBSS. Rs1 showed significantly higher G_q_ signaling than the wildtype 5-HT_4_ receptor when activated by cisapride, zacopride, RS39604, and RS67333 but not by serotonin. G_q_ signaling of Rs1-C-5HT_2C_ chimera was activated by cisapride, zacopride, RS23597, RS39604, and RS67333 and was minimally activated by RO110-0235. GR113808 and GR125487 did not activate G_q_ signaling by any of the three receptors. ***p<0.005, * p<0.05 vs. mock-transfected cells (*t* test). Values are ±SD of three experiments.

Before constructing Rs1-5HT_2C_ chimeras to make a G_q_-signaling RASSL, we assayed inositol phosphate 1 (IP1) accumulation by Rs1 via constitutive or ligand-induced signaling. Rs1 showed no measurable difference in constitutive G_q_ signaling (Figure 5) as compared to mock-transfected cells in the IP1 and calcium mobilization assays. Upon activation by cisapride, zacopride, RS23597, RS39604, or RS67333, Rs1 showed 2–3.5-fold higher G_q_ signaling than the wildtype 5-HT_4_ receptor (p<0.005) (Figure 5). Surprisingly, GR113808, GR125487, and RO110-0235 activated predominately G_s_ (Figure 2) and little G_q_ signaling (Figure 5) by Rs1. These are the same ligands used to selectively activate the G_s_ signaling of Rs1 without activating the G_s_ signaling of the wildtype 5-HT_4_ receptor (Figure 2). Therefore, we could use drugs with distinct chemical structures (Figure S1) to activate G_s_ or G_s_/G_q_ signaling of Rs1. The once controversial use of conformation-specific ligands to alter G-protein coupling and other receptor functions has now been demonstrated in several other GPCRs [25]–[29]. This is the first time that agonist-dependent functional selectivity has been shown in a RASSL.

### Replacing the C-terminus of Rs1 with 5-HT_2C_ increases G_q_ signaling

To make a purely G_q_ signaling RASSL from Rs1, we exchanged the intracellular loops of Rs1 with those of the G_q_-coupled human 5-HT_2C_ receptor. By transferring domains at different junctions of intracellular loops, we made 12 different Rs1-5-HT_2C_ chimeras (Figures S2, S6, S7, S8, S9, S10, S11, S12, S13, S14, S15, S16, S17). To characterize them, we used RS23597 because it activated G_q_ signaling of Rs1 but not the wildtype 5-HT_4_ receptor, as measured by calcium mobilization assays (Figure S3C).

Replacing the second (i2) or third intracellular loop (i3) of Rs1 eliminated both G_s_ and G_q_ signaling (Figure S3). Only the carboxyl chimera (Rs1-C-5-HT_2C_) showed enhanced G_q_ signaling in response to cisapride, zacopride, RS23597, RS39604, and RS67333 (Figure 5), showing that the ligand-induced specificity of signaling was preserved. Constitutive and ligand-induced G_s_ signaling were also largely preserved (Figures S3A, S3B).

These data indicated that i2 and i3 are both necessary for G_s_-coupling of the 5-HT_4_ receptor. They also suggested that the C-terminus of 5-HT_2C_ contains a G-protein coupling domain for G_q_ signaling or that the C-terminus of 5-HT_4_ receptor promotes G_q_ signaling. However, we were unable to completely alter the G-protein preference of this receptor for G_q_ signaling. We amplified the G_q_ signaling of Rs1 by domain swapping the C-terminus with 5-HT_2C_. None of the other 12 chimeras showed enhanced G_q_ signaling, even when multiple internal segments were combined. We did not proceed further with these experiments because substitutions of multiple internal domains also decreased cell-surface expression of the receptors (data not shown). Since G_q_ signaling of Rs1-C-5-HT_2C_ was activated by cisapride, zacopride, and RS23597 but not by serotonin, we named it Rs1.1 (Table 1).

**Table 1:** Controlling the G-protein signaling of Rs1

Receptors	Description	Constitutive signalling G_s_	G- protein signaling		
			G_i_	G_s_	G_q_
5-HT_4_	5-HT_4_	++	–	+++	+
Rs1	5-HT_4_-D^100^A	+++	–	+++	+
Rs1.1	Rs1-C-5-HT_2C_	+++	N/A	+++	++
Rs1.2	Rs1-D^66^A	+	N/A	+	–
Rs1.3	Rs1-i3-5-HT_1A_	–	+	–	N/A

Constitutive signaling and ligand-induced signaling of Rs1 were successfully controlled by point mutations, drug choice, and domain swapping. G_q_ signaling of Rs1 could be activated by zacopride or RS23597 but not GR113808, GR125487 or RO110-0235. The signaling was significantly increased by switching the carboxyl tail (Rs1.1). Attempts to decrease constitutive activity also decreased ligand-induced G_s_ signaling and abolished G_q_ signaling (Rs1.2). Replacing i3 of Rs1 with that of 5-HT_1A_ resulted in a G_i_ coupled RASSL with no G_s/q_ signaling and G_i_ signaling (Rs1.3).

Moreover, when Rs1-C-5-HT_2C_ was activated by cisapride, zacopride, RS23597, RS39604, or RS67333, signaling was 4.5–6.8-fold higher than constitutive signaling (p<0.005). Signaling increased 1.3-fold in response to GR125487 (p<0.05), 1.7-fold in response to GR113808 (p<0.05), and 2.2-fold in response to RO110-0235 (p<0.005). This suggests that the carboxylic tail may play a role in functional selectivity in 5-HT_4_ or 5-HT_2C_ receptors (Figure S3).

### A purely G_s_ signaling RASSL with low levels of constitutive signaling

**Figure 6:**
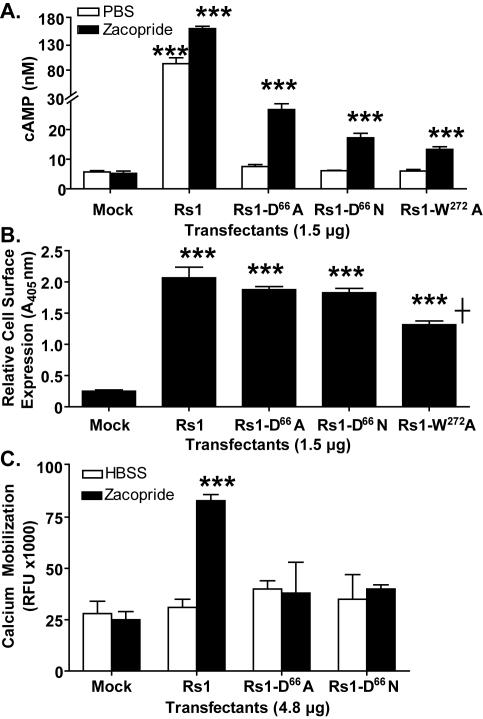
Abolishing the constitutive activity of Rs1 eliminates ligand-induced G_s_ and G_q_ signaling. (A) The D^66^A, D^66^N, and W^272^A mutations each decreased the constitutive signaling of Rs1, as shown by cAMP HTRF HiRange assay. (B) Cell-surface expression levels of Rs1-D^66^A, Rs1-D^66^N, and Rs1 were similar and higher than that of Rs1-W^272^A. _***_ p<0.05 vs. Rs1 (*t* test). (C) The D^66^A or D^66^N mutations also abolished G_q_ signaling of Rs1, as shown by HTRF IP1 assay.

Since the high constitutive activity of the 5-HT_4_-D^100^A mutant causes significant phenotypes in transgenic mice [30] and could not be controlled by inverse agonists [31], we attempted to lower the Rs1 constitutive activity by making additional point mutations. We focused on the D^66^N and W^272^A mutations, which reduce constitutive signaling of the mouse 5-HT_4_ receptor [31], [32]. Rs1-D^66^A, Rs1-D^66^N, and Rs1-W^272^A significantly reduced constitutive signaling (Figure 6A). The cell-surface expression of Rs1-D^66^A and Rs1-D^66^N was similar to that of Rs1 (Figure 6B), so the reduction in constitutive signaling was probably not linked to lower cell-surface expression. Surprisingly, the D^66^A and D^66^N mutations also abolished zacopride-induced G_q_ signaling (Figure 6C). Thus, we created two RASSLs exhibiting pure G_s_ signaling and low constitutive signaling. Unfortunately, the efficacy of the ligand-induced G_s_ response was also significantly compromised, diminishing the utility of these receptors.

### Engineering Rs1 for G_i_ Signaling

**Figure 7:**
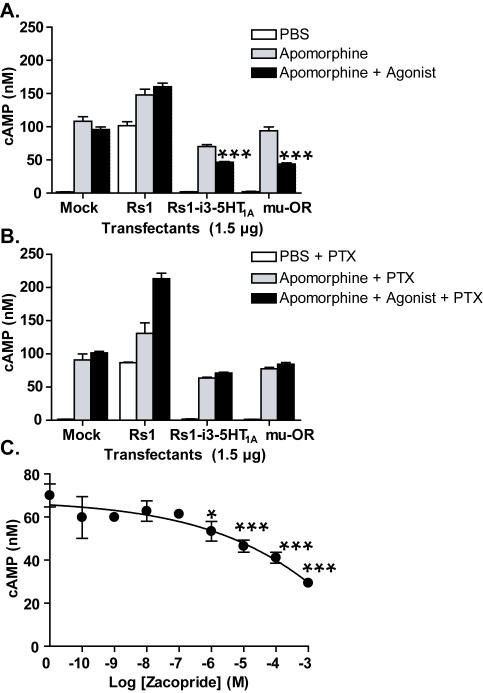
Replacing i3 of Rs1 with that of 5-HT_1A_ results in a receptor, Rs1-i3-5-HT_1A_, with weak G_i _signaling. (A) Rs1-i3-5-HT_1A_ chimera decreased cAMP accumulation. It also showed little constitutive G_s_ signaling, in contrast to Rs1. All HEK293 transfectants were electroporated with 0.6 µg of human dopamine 1 receptor and 1.5 µg of Rs1, Rs1-i3-5-HT_1A_ chimera, or human mu-opioid receptor. The transfectants were stimulated with 10 µM apomorphine (agonist for the dopamine 1 receptor) to increase basal cAMP level in order to observe G_i_ signaling. Rs1 and Rs1-i3-5-HT_1A_ were then stimulated with 10 µM zacopride. The mu-opioid receptor was stimulated with 10 µM DADLE. ***p<0.005, * p<0.05 vs. apomorphine (*t* test). (B) Treatment with pertussis toxin (PTX) abolished the decreased cAMP accumulation of Rs1-i3-5HT_1A_ and mu-opioid receptor, indicating that the decreased cAMP accumulation seen in panel (A) was due to G_i_ signaling. The results are representative of three independent experiments. Values are mean±SD. (C) Rs1-i3-5HT_1A_ required a large amount of zacopride for maximal G_i_ response. The data are representative of two independent experiments. ***p<0.005, *p<0.05 (*t* test), zacopride vs. no treatement.

To engineer a G_i_-signaling RASSL based on Rs1, we replaced its intracellular loops with those of 5-HT_1A_, a G_i_ signaling receptor [33]. Of four Rs1-5HT_1A_ chimeras (Figures 7, S4, S18, S19, S20, S21), only the two containing i2 and i3 from Rs1 were expressed at a level similar to that of Rs1 (Figure S5D). Replacing those loops abolished constitutive and ligand-induced G_s_ signaling at both low and high levels of receptor cDNA (25 ng and 4.8 µg per 5×10^6^ cells) (Figures S5A, S5B). Interestingly, this RASSL showed no evidence of constitutive signaling via the G_i_ or G_s_ pathway. These findings strongly imply that both i2 and i3 are required for G_s_ signaling of Rs1. In addition, activation of the Rs1-i3-5-HT_1A_ chimera with zacopride significantly inhibited cAMP accumulation induced by 10 µM apomorphine (agonist for dopamine 1 receptors) in HEK293 cells co-transfected with 1.5 µg of Rs1 receptor and 0.5 µg of dopamine 1 receptor (per 5×10^6^ cells; Figure S5C and Figure S8A). This inhibition was smaller than that of µ-opioid receptor stimulated by [D-Ala2, D-Leu5]-enkephalin (DADLE). Both responses were abolished by 50 nM pertussis toxin, indicating the involvement of G_i_ signaling (Figure 7B). Unfortunately, the potency (amount of drug needed to reach an effect) of ligand-induced G_i_ signaling was significantly reduced (Figure 7C). While these results are encouraging, future experiments are needed to determine if low potency may be due to nonspecific effects, or will reproduce in other cell types. Since Rs1-i3-5HT_1A_ exhibited G_i_ but not G_s_ signaling, we named it Rs1.3 (Figure 1, Table 1).

## Discussion

### A new series of RASSLs

We report here a new series of RASSLs to study multiple G-protein signaling pathways. Many GPCRs activate multiple G-protein signaling pathways and exhibit a wide range of constitutive signaling activities. Our new RASSLs will help us better study the effect of stimulating canonical signaling pathways (G_s_/G_q_, G_s_, G_i_), with different constitutive signaling using a single receptor backbone and different synthetic agonists. These new RASSLs will also allow a systematic examination of the different functional domains of the 5-HT_4B_ receptor.

These new RASSLs could help us better dissect the physiological significance of constitutive signaling *in vivo*. Constitutive signaling is crucial for many physiological processes and many diseases. Up to 40% of all GPCRs [34], including the 5-HT_4_ receptor [35], show significant constitutive activity, and Rs1 could be a good model for these receptors. Like the mouse 5-HT_4_-D^100^A receptor [22], Rs1 had a higher level of constitutive signaling than the wildtype 5-HT_4_ receptor. Using the tetracycline transactivator (Tet) system, we have already made an Rs1 transgenic mouse in which Rs1 expression is driven by the osteoblast-specific Col1α-1 2.3-kb promoter fragment. These mice exhibited dramatically increased bone formation [30]. These and other findings strongly suggest that constitutive signaling can drive potent phenotypic changes *in vivo*. We found only constitutive G_s_ signaling in Rs1 but did not observe any constitutive G_q_ signaling. It would be interesting to generate new or modify existing RASSLs with increased G_i_ or G_q_ constitutive signaling in the future to examine the effect of both agonist-mediated and constitutive signaling in various physiological processes. Since signaling would only be dependent on expression rather than on circulating hormones, these RASSLs have an intrinsic advantage for studying constitutive signaling when combined with a Tet system.

In addition, the 5-HT_4_ RASSLs have a large set of agonists with different activities that could be useful in future studies. Many of the Rs1 agonists used in this study activated Rs1 with an EC_50_ in the nanomolar range, allowing to us activate Rs1 effectively. Having an abundant selection of ligands for Rs1 helped us find strong evidence of ligand-induced functional selectivity on Rs1. Having a greater variety of drugs to choose from could be valuable for *in vivo* studies. For instance, we could use GR125487 to activate Rs1 while suppressing the basal activity of wildtype 5-HT_4_. Alternatively, we could potentially study Rs1 constitutive signaling by using drugs such as RO116-0086 or RO116-1148 [31] to decrease constitutive signaling of the wildtype 5-HT_4_ receptor but not of Rs1.

Finally, this new series of RASSLs may make it possible to perform *in vivo* studies in which Rs1 is activated with minimal side effects. Since knockout of the 5-HT_4_ receptor does not cause overt side effects [36], treatment with GR113808, GR125487, and R0110-0235 (antagonists and inverse agonists for the wildtype receptor) may have minimal side effects as well. This eliminates the need to knock out the endogenous 5-HT_4_ receptors for most *in vivo* experiments [10]. Since the same agonists can be used to activate all of the RASSLs within this series and therefore+ engage different G-protein signaling pathways (Table 1), we can more easily compare the effect of activating the G_s, _G_s/q_, or G_i_ pathway.

While these new RASSLs may be useful for studying G protein signaling *in vivo*, they have several limitations. Despite significant efforts to screen all available antagonists, we could not identify inverse agonists that would lower constitutive signaling by Rs1. Fortunately, we can use conditional expression systems such as the tetracycline transactivator system to control Rs1 expression and constitutive activation. Although we were successful in finding mutations that reduced Rs1 constitutive signaling these same mutations adversely affected the agonist mediated signaling (Rs1.2), suggesting that the D^66^N and W^272^A mutations are critical for all aspects of G_s _signaling. Other mutations could be explored that may provide a more optimal reduction in G_s_ basal activity without affecting ligand-induced receptor activation. In addition, the relatively small G_i _signaling of Rs1.3 can only be activated by a relatively high concentration of zacopride, thus limiting its usefulness *in vivo*.

### Insights into G-protein signaling by Rs1

Our study yielded insights into the G-protein selectivity and functional selectivity (differential effects of ligands on the same receptor) of Rs1. Of the 12 Rs1-5-HT_2C_ chimeras that are expressed on the cell surface, none of the i2 or i3 chimeras showed any G_s_ or G_q_ signaling. Evidently, these intracellular loops of Rs1 are crucial for signaling via those pathways. The importance of i2 and i3 for G_s_ signaling of Rs1 is further supported by the lack of G_s_ signaling by the Rs1-i2-5-HT_1A_ and Rs1-i3-5-HT_1A_ chimeras. This is the first study showing the importance of i2 and i3 in both G_s_ and G_q_ signaling of the human 5-HT_4_-D^100^A receptor.

We also found that i3 domain swapping abolished all G_s_ signaling and enabled Rs1 to stimulate G_i_ signaling of 5-HT_1A_. The role of i2 and i3 in the G_i_ signaling of 5-HT_1A_ receptor has been extensively reported. The entire N-terminus [37] and C-terminus of i2 [38] of 5-HT_1A_ are thought to be sufficient to support G-protein coupling, but not signaling. On the other hand, the N-terminus [39] and C-terminus of i3 of 5-HT_1A_ [40], [41] seem to be essential for the G_i_ signaling of 5-HT_1A_. In fact, replacing the N-terminus of the i3 of the α_2_-adrenergic receptor with that of 5-HT_1A_ resulted in a chimera that signals like a 5-HT_1A_ receptor when stimulated by a α_2_-adrenergic receptor agonist [42]. Since Rs1-5HT_1A_ chimeras with multiple internal domains replaced are not significantly expressed on the cell surface (data not shown), it may be difficult to further improve the potency of the Rs1.3 using our current approach. We hypothesize that replacing the N- and C-terminal portions of i2 and i3 instead of the whole i2 and i3 loops may increase the potency of Rs1.

We also showed that various drugs can differentially activate G-protein signaling of Rs1. Functional selectivity has been reported for many receptors. It led to divergent fates of internalization for the dopamine D_1_ receptor [25], various binding specificities for gonadotropin-releasing hormone receptors, and different levels of activation of G proteins for the β_2_-adrenergic [26], mu-opioid [27], dopamine D_2_ [28], and human 5-HT_2A_ [29] receptors.

The indoleamine derivatives GR113808, GR125487, and RO110-0235 did not fully activate G_q_ signaling of Rs1, Rs1.1, or the 5-HT_4_ receptor. On the other hand, the benzamide derivatives cisapride, zacopride, RS23597, RS39604, and RS67333 activated the G_q_ signaling of Rs1. These findings may reflect distinct conformational changes caused by indoleamine and benzamide derivatives.

The possibility of functional selectivity is further supported by the results obtained with Rs1-C-5HT_2c_ and Rs1 point mutants (Rs1-D^66^A and Rs1-D^66^N). The D^100^A mutation and replacement of the C-terminus amplified G_q_ signaling by the 5-HT_4_ receptor. The addition of D^66^A and D^66^N abolished G_q_ signaling. Since D^100^A is located in the binding pocket of the 5-HT_4_ receptor, this mutation in Rs1 may have changed the configuration of the binding pocket, making the receptor more susceptible to G_q_ activation by zacopride and RS23597. This response was even more pronounced when the D^100^A mutation was combined with domain swapping of the C-terminus with that of 5-HT_2C_. Thus, it is reasonable to hypothesize that these changes modified the ligand-selective receptor conformation [5], changing the receptor susceptibility to functional selectivity.

### Conclusions

Our studies with Rs1 provide a proof-of-concept for making a series of RASSLs with different signaling properties. Recently, Armbruster *et al.* made a series of RASSLs based on the muscarinic M3 and M4 receptors, which have low constitutive activity. These RASSLs each couple different G-protein signaling pathways and can be activated by clozapine-N-oxide, an inert ligand with high bioavailability [16]. These RASSLs nicely complement our Rs1 RASSLs with varying constitutive activity. In addition, we predict that some RASSLs with the same canonical G-protein signaling (G_s_, G_i_, or G_q_) will have different *in vivo* phenotypes due to noncanonical signaling. This growing collection of RASSLs will greatly facilitate our efforts to understand the physiological significance of the inherent signaling diversity of GPCRs.

An ideal series of RASSLs would have receptors with different combinations of low and high basal signaling, with robust ligand-induced effects for each major pathway, and potent inverse agonists. Although we have not achieved this goal with the Rs1 series, we are hopeful that it can be achieved with other receptors in the future. Indeed, RASSLs based on the muscarinic receptors [16] show great promise, as there are naturally occurring, or published mutants of the muscarinic reports that activate each of the major G protein signaling pathways.

## Materials and Methods

### Constructing human 5-HT_4_ mutant cDNA and Rs1-5-HT_1A_, and Rs1-5-HT_2C_ chimeras

The human 5-HT_4_ receptor cDNA (a gift from Dr. Bryan Roth, University of North Carolina) was used in all experiments. To improve expression and allow detection of the receptor, we added a signal peptide from influenza hemagglutinin [43] and a FLAG epitope (DYKDDDDA) at the N-terminus. 5-HT_4_ was then subcloned by PCR; the primers, ATCGATCGgcggccgcGTGAGCAAGGGCGAGGAGCTGTTC and ATCGATCG gcggccgcCTAAGTGTCACTGGGCTGAGCAGCC, were inserted into the Not1 restriction site (gcggccgc) of the pUNIV-5-HT_2C_-INI plasmid to replace the 5-HT_2C_-INI (a gift from Dr. Bryan Roth) in frame with the signal peptide and the FLAG epitope. The receptor was then mutated (D^100^A) with a Quick-Change site-directed mutagenesis kit (Stratagene, La Jolla, CA) with primer GTCTTGTTCGGACATCTCTG**gcc**GT CCTGCTCACAACGGCAT-CG (Figures 1, S2). The following mutant sense primers were used: 5-HT_4_-D^66^A, TTCATTGTATCTCTTGCTTTTGCG**gca**CTGCT GGTTTCGGTGCTGGT-GATG; 5-HT_4_-D^66^N, TTCATTGTATCTCTTGCTTTTGCG**aac**C TGCTGGTTTCGGTGCT-GGTGATG; and 5-HT_4_-W^272^A, GTTGCTTCTGCCTCTGCT GGGCGCCA**gcc**TTTGTCA-CCAATATTGTGG. The sense primer used to replace the carboxyl chimera for Rs1.1 (Table 1) was AGTTACTCTTCC**gcg**GCCGCGAATTCAG TGGATCCACTAGTAAC. The Rs1-5-HT_1A_ and Rs1-5-HT_2C_ chimeras were made by PCR fusion. The mutations are indicated by boldface, lower-case letters.

### HEK293 maintenance and electroporation

Early-passage (≤20) HEK293 cells were maintained in high-glucose DMEM (Invitrogen, Carlsbad, CA) supplemented with sodium pyruvate (Invitrogen) and 10% Fetalplex (Gemini Bio-Products, West Sacramento, CA). Receptors were electroporated into HEK293 cells as described [44]. The electroporated cells were reconstituted into a suspension using DMEM with 10% heat-inactivated, dialyzed fetal bovine serum (Thermo-Fisher Scientific, Logan, UT). The transfection efficiency was monitored by flow cytometry, and the cell-surface expression of the receptor was determined by FLAG ELISA (enzyme-linked immunosorbent assay) the next day.

### Drugs

5-HT, isoproterenol, and 3-isobutyl-1-methylxanthine (IBMX) were purchased from Sigma-Aldrich (St. Louis, MO). Cisapride, zacopride, GR113808, GR1254875, RS23597-190 HCl, RS39604 HCl, and RS67333 HCl were from Tocris (Bristol, UK). Human parathyroid hormone peptide (amino acids 1–34) was from Bachem Biosciences (King of Prussia, PA). RO110-0235 was generously donated by Renee Martin (Roche, Palo Alto, CA).

### Measuring cell-surface expression by FLAG ELISA

Cell-surface receptor expression was measured with a FLAG ELISA as described [45]. Cells seeded in poly-D-lysine-coated 96-well plates were fixed with 100 µl of 4% paraformaldehyde for 10 min at room temperature, washed, and stained with 100 µl of staining buffer (DMEM, 10% FBS, and 1 mM CaCl_2_) containing anti-FLAG M1 antibody (1∶1000; Sigma-Aldrich) for 1 h at 25°C. The samples were washed three times with wash buffer (PBS and 1 mM CaCl_2_) and stained with 100 µl of staining buffer with rat anti-mouse IgG antibody conjugated with horseradish peroxidase (1∶1000; Bio-Rad Laboratories, Hercules, CA). After 30 min, the samples were washed with wash buffer, placed on a rocker for 10 min, and washed again. This process was repeated two more times. Then, 2,2-azino-bis(3-ethylbenzthiazoline-6-sulfonic acid) liquid substrate (200 µl; Sigma-Aldrich) was added to the samples. After rocking for 30 min, 200 µl of the substrate was transferred to new 96-well plates, and optical density was measured with Victor 3 (PerkinElmer, Waltham, MA) at 405 nm. All samples contain three replicates, and all experiments were repeated at least three times.

### Measurement of cAMP production in intact cells

To improve assay consistency and minimize pipetting error in the 384-well plates, we modified the high-range HTRF assay (CisBio International, Bagnols-sur-Cèze, France) by seeding, stimulating, and lysing the cells in 96-well plates and using the lysate instead of live cells to determine cAMP production. The remainder of the analysis was performed according to the manufacturer's instructions

### G_i_ Assay

G_i_ signaling was examined in cells transfected with 1.5 µg of receptor cDNA, 0.6 µg of human dopamine 1 receptor cDNA, and pcDNA3 (up to 6 µg). The co-transfectants were stimulated first with 100 µl of KRBG buffer containing IBMX for 10 min at room temperature and then with 50 µl of PBS containing 10 µM apomorphine (agonist for the dopamine 1 receptor) and 10 µM zacopride for 10 min at 37°C. The cells were lysed in 50 µl of lysis buffer, and 5 µl of lysate was used in the HiRange HTRF assay.

### Fluorometric imaging plate reader assay to measure calcium mobilization

To measure calcium mobilization, 4.8 µg of receptor cDNA, 0.6 µg of DsRed plasmid, and 0.6 µg of human bombesin receptor cDNA were electroporated into 5×10^6^ HEK293 cells as above [44]. Hank's balanced salt solution (10 ml) with 20 mM HEPES, 0.25 mM probenecid acid (Sigma-Aldrich), and 2% pluronic acid (Sigma-Aldrich) was added to each bottle of Calcium 4 (Molecular Devices, Sunnyvale, CA), and 100 µl of the resulting solution was added to each well for 1 h at 37°C before measurement. Assays were performed with a FLEX Station (Molecular Devices), with excitation of 485 nm, emission of 525 nm, and cut-off of 515 nm, as recommended by the manufacturer.

### Determination of IP1 production in intact cells

A modified version of the IP1 protocol was used (CisBio International). HEK293 cells were washed once with calcium-free PBS and dissociated from flasks with cell dissociation buffer (Invitrogen). Cells (5×10^6^) were electroporated as described above. Then, 10^5^ cells were placed in DMEM supplemented with 10% decomplemented, dialyzed against FBS, and seeded onto 96-well plates coated with poly-D-lysine. The next day, the cells were stimulated with agonists in 50 µl of 1× stimulation buffer for 30 min at 37°C and lysed for 10 min with 9 µl of lysis/detection buffer. Then, 14 µl of lysate was added to 384-well plates and subjected to High-Range HTRF assay as described above, except that 3 µl of cAMP-d2 and anti-cAMP-cryptate solution were added to each well.

### Data analysis

cAMP and IP1 values were analyzed with GraphPad Prism 4 (GraphPad Software, San Diego, CA). Calcium mobilization results were analyzed with SoftMax Pro v5 (Molecular Devices). Statistical significance was determined with paired Student's *t* tests.

## Supporting Information

Figure S1 Chemical structures of the compounds used in the study. All the chemicals used in the experimental are shown.
(1.92 MB EPS)

Figure S2 Rs1-5-HT2C chimeras. All modifications made to Rs1 (Figure 1). Red lines indicate the junctions of chimeras. The amino acids exchanged are shown by the amino acid alignment.
(12.17 MB EPS)

Figure S3 The second and third intracellular loops (i2 and i3) of Rs1 are crucial for Gs and Gq signaling. (A, B) Rs1-5-HT2C chimeras with swaps of i2 and i3 could no longer process Gs signals, at either 25 ng or 4.8 µg of receptor cDNA per 5×106 HEK293 cells. (C) Gq signaling of Rs1 was abolished when i2 and i3 of Rs1 were replaced with those of 5-HT2C. The Gq signaling was measured by calcium mobilization assay. (D) Only chimeras with a single domain swap were expressed on the cell surface. The results represent three independent experiments. All figures were representative of three independent experiments.
(2.03 MB EPS)

Figure S4 Rs1-5-HT1A chimeras. All modifications were made on Rs1 (Figure 1). Red lines indicate the junctions of chimeras. The amino acids exchanged are shown by the amino acid alignment.
(3.82 MB EPS)

Figure S5 Replacing the second or third intracellular loop (i2 or i3) of Rs1 with 5-HT1A alters G protein signaling. (A, B) Rs1-5-HT1A chimeras with swaps of the second (i2) and third intracellular (i3) loops no longer signal via the Gs pathway, regardless of whether cells were transfected with 25 ng or 4.8 µg of receptor cDNA per 5×106 HEK293 cells. This suggests that the second and third intracellular loops are crucial for acute Gs signaling of Rs1. ***p<0.001 vs. mock transfected (t test). (C) Replacing i3 of Rs1 resulted in a Gi signaling receptor. All HEK293 transfectants were electroporated with 0.6 µg of the human dopamine 1 receptor and 1.5 µg of Rs1, Rs1-5HT1A chimeras, or the mu-opioid receptor. Rs1 and Rs1-5HT1a chimeras were treated with 10 µM apomorphine (an agonist for dopamine 1 receptor) or with 10 µM apomorphine and 10 µM zacopride. Transfectants with 0.6 µg of the human dopamine 1 receptor and 1.5 µg of the mu-opioid receptor served as positive controls. 10 µM DADLE was used in place of zacopride to stimulate mu-opioid receptors. ***p<0.005, *p<0.05 vs. apomorphine (t test). (D) Rs1, (i1, i2 and i30 were expressed on the cell surface. Cell-surface expression and calcium mobilization of the chimeras were examined at 4.8 µg of receptor DNA per 5×106 HEK293 cells. The results are representative of three independent experiments. Values are mean±SD. ***p<0.001 vs. mock transfected (t test).
(1.67 MB EPS)

Figure S6 High-resolution representation of Rs1-5HT1A and Rs1-5HT2C chimeras. All modifications made to Rs1 (Figure 1). Red lines indicate the junctions of chimeras. The amino acids exchanged are shown by the amino acid alignment.
(2.98 MB EPS)

Figure S7 High-resolution representation of Rs1-5HT1A and Rs1-5HT2C chimeras. All modifications made to Rs1 (Figure 1). Red lines indicate the junctions of chimeras. The amino acids exchanged are shown by the amino acid alignment.
(2.96 MB EPS)

Figure S8 High-resolution representation of Rs1-5HT1A and Rs1-5HT2C chimeras. All modifications made to Rs1 (Figure 1). Red lines indicate the junctions of chimeras. The amino acids exchanged are shown by the amino acid alignment.
(3.00 MB EPS)

Figure S9 High-resolution representation of Rs1-5HT1A and Rs1-5HT2C chimeras. All modifications made to Rs1 (Figure 1). Red lines indicate the junctions of chimeras. The amino acids exchanged are shown by the amino acid alignment.
(2.99 MB EPS)

Figure S10 High-resolution representation of Rs1-5HT1A and Rs1-5HT2C chimeras. All modifications made to Rs1 (Figure 1). Red lines indicate the junctions of chimeras. The amino acids exchanged are shown by the amino acid alignment.
(2.99 MB EPS)

Figure S11 High-resolution representation of Rs1-5HT1A and Rs1-5HT2C chimeras. All modifications made to Rs1 (Figure 1). Red lines indicate the junctions of chimeras. The amino acids exchanged are shown by the amino acid alignment.
(2.99 MB EPS)

Figure S12 High-resolution representation of Rs1-5HT1A and Rs1-5HT2C chimeras. All modifications made to Rs1 (Figure 1). Red lines indicate the junctions of chimeras. The amino acids exchanged are shown by the amino acid alignment.
(3.00 MB EPS)

Figure S13 High-resolution representation of Rs1-5HT1A and Rs1-5HT2C chimeras. All modifications made to Rs1 (Figure 1). Red lines indicate the junctions of chimeras. The amino acids exchanged are shown by the amino acid alignment.
(2.99 MB EPS)

Figure S14 High-resolution representation of Rs1-5HT1A and Rs1-5HT2C chimeras. All modifications made to Rs1 (Figure 1). Red lines indicate the junctions of chimeras. The amino acids exchanged are shown by the amino acid alignment.
(3.00 MB EPS)

Figure S15 High-resolution representation of Rs1-5HT1A and Rs1-5HT2C chimeras. All modifications made to Rs1 (Figure 1). Red lines indicate the junctions of chimeras. The amino acids exchanged are shown by the amino acid alignment.
(2.99 MB EPS)

Figure S16 High-resolution representation of Rs1-5HT1A and Rs1-5HT2C chimeras. All modifications made to Rs1 (Figure 1). Red lines indicate the junctions of chimeras. The amino acids exchanged are shown by the amino acid alignment.
(3.00 MB EPS)

Figure S17 High-resolution representation of Rs1-5HT1A and Rs1-5HT2C chimeras. All modifications made to Rs1 (Figure 1). Red lines indicate the junctions of chimeras. The amino acids exchanged are shown by the amino acid alignment.
(2.96 MB EPS)

Figure S18 High-resolution representation of Rs1-5HT1A and Rs1-5HT2C chimeras. All modifications made to Rs1 (Figure 1). Red lines indicate the junctions of chimeras. The amino acids exchanged are shown by the amino acid alignment.
(3.00 MB EPS)

Figure S19 High-resolution representation of Rs1-5HT1A and Rs1-5HT2C chimeras. All modifications made to Rs1 (Figure 1). Red lines indicate the junctions of chimeras. The amino acids exchanged are shown by the amino acid alignment.
(2.96 MB EPS)

Figure S20 High-resolution representation of Rs1-5HT1A and Rs1-5HT2C chimeras. All modifications made to Rs1 (Figure 1). Red lines indicate the junctions of chimeras. The amino acids exchanged are shown by the amino acid alignment.
(3.40 MB EPS)

Figure S21 High-resolution representation of Rs1-5HT1A and Rs1-5HT2C chimeras. All modifications made to Rs1 (Figure 1). Red lines indicate the junctions of chimeras. The amino acids exchanged are shown by the amino acid alignment.
(3.03 MB EPS)
